# Debut of Crohn's disease in a child desensitised to cow’s milk

**DOI:** 10.1186/2045-7022-5-S3-P54

**Published:** 2015-03-30

**Authors:** Karolina Esponda-Juárez, Victoria Fuentes-Aparicio, Gerardo Prieto Bozano, Mónica Rodríguez-Álvarez, Eva Pescosolido, Montserrat Fernández-Rivas

**Affiliations:** 1 Allergy Department, Hospital Clínico San Carlos, Madrid, Spain; 2 Gastroenterlogy Department, Hospital Universitario La Paz, Madrid, Spain

## 

Specific oral immunotherapy (OIT) is a proven therapeutic option in children with persistent food allergy. Several cases of eosinophilic esophagitis have been reported during the long term follow-up, but not inflammatory bowel diseases. The role of food allergy in the pathogenesis of these diseases is unknown.

We present an 11 year-old girl who developed cow's milk allergy (CMA) at the age of 5 months followed by mild asthma in her early childhood. Due to persistent CMA she started cow milk OIT at the age of 8. After a long induction phase - 10 months - in which she presented repeated systemic reactions and abdominal pain, tolerance of 200 ml of cow's milk was reached, and serum levels of cow's milk specific IgE and its components decreased (pre-OIT whole milk sIgE 97.9 kUI_A_/L, casein 97.5 kUI_A_/L; post-OIT whole milk sIgE 27.2 kUI_A_/L, casein 22.7 kUI_A_/L). After 4 months of daily maintenance treatment, she complained about recurrent abdominal pain related to any food ingestion. A pediatric gastroenterologist ruled out the diagnosis of eosinophilic esophagitis, and she was treated with proton pump inhibitors during 18 months while maintaining daily cow’s milk intake. Although the patient improved partially, abdominal pain was not solved. At age 11 an emergency appendectomy was performed because of an acute abdomen caused by a suspected appendicitis. She was advised to maintain a milk-free diet at discharge. One month later she was readmitted for abdominal pain and diarrhea with weight loss. She underwent an exploratory laparotomy with ileum resection upon obtaining macroscopic evidence of Crohn's disease and pathological results which confirmed the diagnostic. Treatment with corticosteroids, azathioprine and 3 cycles of infliximab was initiated, yielding a good response. At present her symptoms are under control using azathioprine 50 mg daily, while tolerating foods containing trace amounts of cow’s milk.

In conclusion, we present a case of a girl who developed Crohn’s disease during the maintenance phase of cow milk OIT. At present we have no rationale for the role of OIT in the development of her Crohn’s disease.

## Consent

Written informed consent was obtained from the parent or guardian of the patient for publication of this abstract and any accompanying images. A copy of the written consent is available for review by the Editor of this journal.

